# Polyurethane/Red Mud Composites with Flexibility, Stretchability, and Flame Retardancy for Grouting

**DOI:** 10.3390/polym10080906

**Published:** 2018-08-12

**Authors:** Chunjing Zhang, Bo Shuai, Xuefeng Zhang, Xinxin Hu, Hui Zhang, Yuanheng Jia, Zhengpeng Yang, Xuemao Guan

**Affiliations:** 1 Institute of Materials Science and Engineering, Henan Polytechnic University, Jiaozuo 454000, China; zcj@hpu.edu.cn (C.Z.); shuaibohpu@163.com (B.S.); xfzhang2018@163.com (X.Z.); xinxinhu2015@163.com (X.H.); yhjia@163.com (Y.J.); guanxuemao@163.com (X.G.); 2 School of Energy and Engineering, Henan Polytechnic University, Jiaozuo 454000, China

**Keywords:** grouting material, polyurethane, red mud, flexibility, stretchability

## Abstract

Flexibility, stretchability, and flame retardancy are of ever increasing importance in constructing grouting materials. Herein, a simple and effective strategy to make organic-inorganic composite grouting material in a “flexible, stretchable, and flame retardant” way was based on the excellent synergistic interactions among polyurethane prepolymer, red mud, polyethylene glycol, and trimethylolpropane. The resultant polyurethane/red mud composite grouting material with three-dimensional network structure presented a favorable flexibility, desirable compressive strength of 29.2 MPa at 50% compression state, and a good elongation at 15.1%. The grouting material was mainly composed of amorphous polyurethane and crystalline red mud, and its probable formation mechanism was reaction of prepolymer with H_2_O, polyethylene glycol and trimethylolpropane under vigorous stirring in the presence of catalyst. Furthermore, the grouting material possessed favorable thermal stability, flame retardancy and repairment performance for roadway cracks. This work may open a simple and convenient avenue for the massive engineering application of red mud and preparation of flexible organic-inorganic hybrid grouting material.

## 1. Introduction

Grouting, which is an effective means of sealing water, stabilizing ground, and repairing cracks, is widely used in civil and mining engineering [1,2,3]. Grouting materials, as the core of grouting technology, play an important role in modern grouting projects. At present, grouting materials with flexibility, stretchability, and flame retardancy are becoming increasingly important due to the requirement of practical construction.

Generally, the currently available grouting materials can be mainly divided into inorganic and organic grouting materials. The inorganic grouting material mainly includes cement [4,5], waterglass [6,7], and clay [8]. The organic grouting materials such as polyurethane [9,10,11], epoxy resin [12], acrylamide [13], methacrylate [14], acrylate [15], and lignin [16] have been extensively explored as well. Among the aforementioned grouting materials, polyurethane has attracted widespread attention owing to its good flexibility, high permeability, strong adhesion, and easy manipulation [17,18,19], and is regarded as one of the most promising candidates. However, polyurethane grouting material still suffers from high price, flammability, poor barrier property, and thermal stability [20,21], which limits its practical applications. Thus, finding an effective and cost-efficient way to achieve polyurethane grouting materials with improved performance is highly desirable.

Inorganic modification is an important strategy in the performance improvement of polyurethane grouting materials. Recently, nanosilica and waterglass have been used to combine with polyurethane in order to improve the performance of grouting materials. Polyurethane/nanosilica hybrid for grouting was prepared though two-step polymerization, and its mechanical properties and thermal stability could be greatly improved [22]. Polyurethane/waterglass grouting materials with flame resistance, low cost, high thermal stability and mechanical properties have also been developed via a facile room-temperature cured process, and presented a satisfactory performance towards grouting reinforcement [23,24,25]. These reports stimulate further research and development of novel preparation methods for polyurethane-based organic-inorganic grouting materials. Red mud, known as a waste product generated in the industrial production of aluminum, cannot be easily disposed, and therefore causes great pollution to the environment [26]. Many efforts have been made in relation to the recycling of waste red mud. Catalysts, radiopaque materials, and adsorbents derived from red mud have been proposed and investigated [27,28,29,30,31]. These studies present good results, but yet are far from meeting the large-scale application of red mud. Therefore, the exploration of massive application of red mud is quite necessary and very challenging. So far, very little has been published in the literature on red mud for grouting. Thus, it is expected that integrating red mud and polyurethane into composite grouting material may make it possible to realize massive engineering application of red mud.

Here, a polyurethane/red mud composite (PRC) grouting material with flexibility, stretchability and flame retardancy was prepared successfully via a simple and effective strategy. The microstructure and property of the resultant grouting material were explored in detail, and its possible formation mechanism was also proposed.

## 2. Experimental

### 2.1. Materials

The prepolymer (16.7 wt % NCO, 420 mPa·s) synthesized with diphenyl methane diisocyanate and polyether polyol was purchased from Shandong INOV Polyurethane Co., Ltd. (Zibo, China) The red mud was mostly composed of Fe_2_O_3_, Al_2_O_3_, SiO_2,_ and TiO_2_ particles sampled from Zhongzhou Branch of China Aluminum Co., Ltd. (Jiaozuo, China) Polyethylene glycol (PEG, molecular weight 600) was supplied from Jiangsu Haian Petroleum Chemical Factory (Haian, China). Trimethylolpropane (TMP) was obtained from Guangzhou SPT Chemical Co., Ltd. (Guangzhou, China). Emulgator (Alkylphenol polyoxyethylene, OP-9, 93 mg KOH/g) was supplied by Shanghai Sinopharm Chemical Reagent Co., Ltd. (Shanghai, China). Catalyst (*N*-ethylmorpholine, 99%) was purchased from J&K Scientific Ltd. (Shanghai, China). All other chemicals were of analytical grade and purchased from Shanghai Sinopharm Chemical Reagent Co., Ltd. (Shanghai, China).

### 2.2. Preparation of PRC Grouting Material

The PRC was prepared via a facile room-temperature-cured process. The effect of main components on the performance of PRC was investigated (Supplementary Material), and the optimized preparation process was described as follows. Briefly, water, red mud, PEG, TMP, emulgator and catalyst with weight ratio of 19:56.5:21:2.5:0.6:0.4 were successively added into a 400 mL plastic cup and mixed for 20 min at 7000 rpm using a mechanical stirrer, denoted as component A. The prepolymer was introduced into another plastic container, denoted as component B. Subsequently, component A and component B with equal volume were mixed under room temperature. After vigorous stirring for 20 s, the resultant suspension was fully cured for tests.

### 2.3. Characterization

Section microstructures of the samples were performed in s scanning electron microscope (SEM, Merlin Compact, Carl Zeiss NTS Gmbh, Oberkochen, Germany), the sample surface was clad with gold before scanning. Infrared spectra were recorded on Nicolet 200SXV Fourier transform infrared (FTIR) spectrometer using a KBr wafer. X-ray diffraction (XRD) patterns were recorded on a Bruker-AXS D8 (Karlsruhe, Germany) X-ray diffractometer system operating with a Cu-Kα radiation. The thermo gravimetric and differential thermal analysis (TG-DTA) data were obtained between 35 and 700 °C with the HCT-1 (Beijing Henven Scientific Instument Factory, Beijing, China) thermal analyzer system at a heating rate of 10 °C/min, under air atmosphere.

### 2.4. Mechanical Measurements

All the tensile and compressive strength measurements were conducted using an electron omnipotence experiment machine (Model WDW-20, Jinan Hengruijin Instrument Equipment Co., Ltd., Jinan, China) at room temperature. The tensile tests were done on the strip-shaped samples with dimensions 100.0 × 20.0 × 3.0 mm^3^ with a constant loading rate of 5 mm/min. The compressive tests were performed on samples with dimensions 40.0 × 40.0 × 40.0 mm^3^ with a constant loading rate of 1 mm/s.

## 3. Results and Discussion

**Figure 1:**
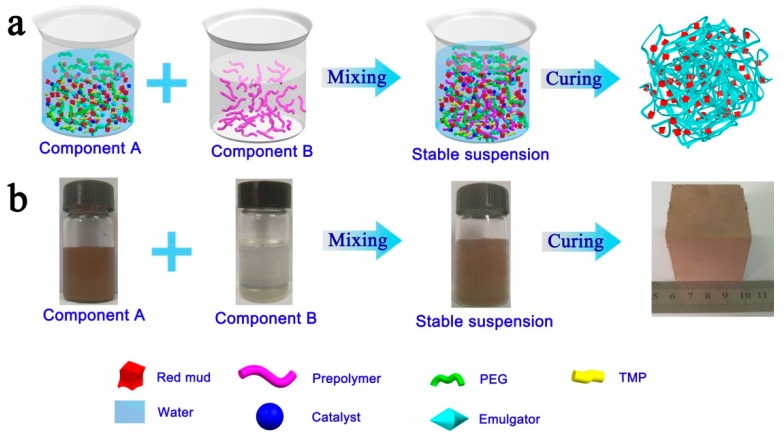
(**a**) Schematic illustration of the fabrication process of polyurethane/red mud composite (PRC). (**b**) Digital photographs of component A, component B, stable suspension, and cured PRC.

The overall preparation procedure for PRC is schematically illustrated in Figure 1. At first, red mud, PEG, TMP, emulgator, and catalyst were added into water under continuous mechanical stirring to form a homogenous suspension, denoted as component A. The colorless prepolymer was used as component B. Next, component A and component B were mixed together simply at 7000 rpm using a mechanical stirrer, and then a stable suspension was obtained. Finally, a square PRC specimen was formed via a room-temperature-cured process. The dense and smooth-faced PRC reveals a three-dimensional network structure (Figure 1a). Because of the simple and readily accessible synthesis process, PRCs with desired shapes such as cube and cuboid were facilely prepared (Figure 1b).

**Figure 2:**
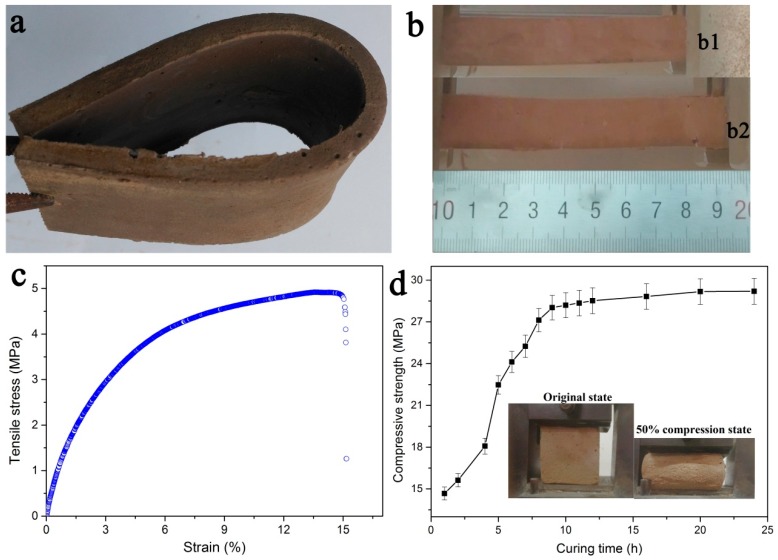
Flexible and mechanical properties of cured PRC. (**a**) A photograph of bending PRC specimen. (**b**) Uniaxial tension test of cured PRC: (**b1**) original state and (**b2**) tensional state. (**c**) Typical stress-strain curve of specimen during the stretching process. (**d**) The compressive strength as a function of curing time. Average of three measurements (mean ± S.D.) The inset photographs display the original and 50% compression status of cured PRC.

The obtained PRC exhibited the intriguing flexibility and good resistance to deformation as shown in Figure 2a. Bending had no damage to the grouting material specimen. Figure 2b presents the original (b1) state and tensional state (b2) of the PRC. The PRC specimen could be stretched by the application of a relatively small force. The tensile stress and strain curve of PRC was shown in Figure 2c, and the fracture strength and corresponding strain of the PRC were 4.8 MPa and 15.1%, respectively. Figure 2d shows the compressive strength of PRC at 50% compression state as a function of curing time. The compressive strength almost linearly increased with the curing time up to nine hours, and then increased slowly to reach a stable value (29.2 MPa). Remarkably, even after compression for 50%, the PRC had no obvious cracks and kept a satisfactory compressive strength, and could almost recover to its initial state, further suggesting the good mechanical strength and flexibility of PRC.

**Figure 3:**
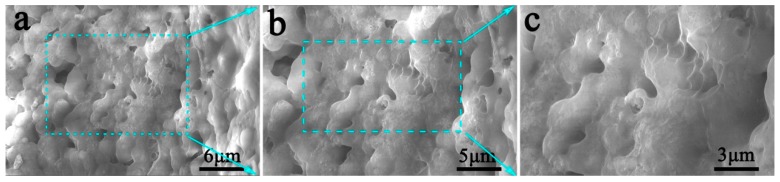
Cross-sectional SEM images of cured PRC. (**a**) Low magnification, (**b**,**c**) High magnification.

The favorable flexibility, stretchability, and mechanical strength of PRC can be closely related to the microscopic structure of the PRC. Figure 3 shows the cross-sectional SEM images of cured PRC, the fracture surface was compact and free from appreciable cracks. From the enlarged SEM microscopy (Figure 3c), it could be clearly observed that the organic phases in PRC were interconnected with each other, forming a network to unite the grouting material into an integrated whole, which accounted for the flexibility and stretchability of PRC. Note that some micron-sized particles were uniformly distributed and tightly wrapped into organic phase, forming an organic-inorganic hybrid composite, which played a crucial part in the relatively high mechanical strength of PRC. Furthermore, we found that a small number of small and irregular pores still existed in PRC specimen due to the CO_2_ production in the curing process of PRC [24]. The SEM observation reveals an integrated and flexible structure of PRC, indicating the excellent reactions among various components of grouting material, which is favorable for its mechanical performance.

**Figure 4:**
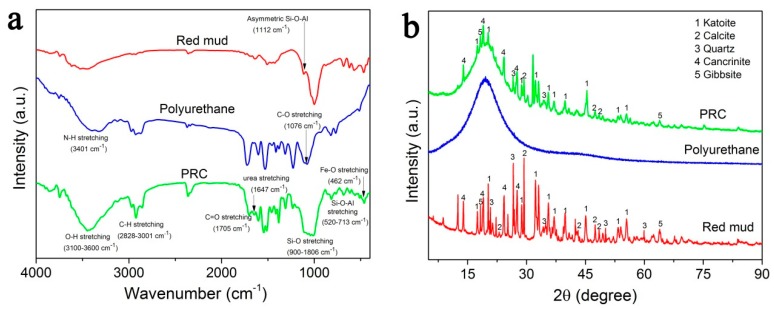
FTIR (**a**) spectra and XRD (**b**) patterns of red mud, polyurethane and PRC.

The chemical structure and composition of PRC were confirmed by FTIR and XRD, and for a comparison, the FTIR spectra and XRD patterns of polyurethane and red mud were also analyzed. As shown in Figure 4a, all characteristic bands of red mud and polyurethane appeared in the FTIR spectrum of PRC, as expected. For example, N-H stretching at 3401 cm^−1^, asymmetric C-H stretching at 2828–3001 cm^−1^, carboxylic C=O stretching at 1705 cm^−1^, C-O stretching at 1076 cm^−1^, bending vibration of aromatic and alkene moieties at 720–869 cm^−1^, O-H stretching at 3100–3600 cm^−1^, asymmetric Si-O-Al stretching at 1112 cm^−1^, Si-O stretching at 900–1086 cm^−1^, Si-O-Al stretching at 520–713 cm^−1^, and Fe-O stretching at 462 cm^−1^. In addition, note that a new weak band corresponding to urea linkage appeared at 1647 cm^−1^ due to the reaction between NCO groups in prepolymer and H_2_O molecules. Figure 4b presents XRD patterns of red mud, polyurethane and PRC, the representative peaks of PRC matched well with the characteristic peaks of polyurethane and red mud. The comparatively broad diffraction peak at the region of 2θ = 10–30° corresponds to the characteristic peak of polyurethane, the relatively strong and narrow diffraction peaks at 2θ = 17.5°, 20.1°, 28.6°, 32.2°, 35.3°, 36.9°, 53.3°, and 55.5° can be perfectly indexed to the typical crystal planes of katoite, the diffraction peaks at 2θ = 29.1°, 47.9°, and 48.4° can be generally considered as the characteristic peak of calcite, the diffraction peaks at about 2θ = 26.5° and 34.4° refer to quartz, the diffraction peaks at 2θ = 13.8°, 24.0°, and 27.4° can be assigned to those XRD patterns of cancrinite and the major peaks of gibbsite appear at 2θ = 18.1° and 63.7°. Notably, the diffraction peaks corresponding to katoite, calcite, quartz, cancrinite, and gibbsite are relatively narrow, suggesting a crystalline structure. The FTIR and XRD analyses indicate that the main components of cured PRC are amorphous polyurethane and crystalline red mud, further confirming the viability of the fabrication of PRC.

According to the analysis from SEM, FTIR, and XRD, the possible formation mechanism of PRC can be described by the following. During the mixing and curing processes, the interface tension between red mud slurry and the organic matrix can be reduced by the emulgator OP-9 and vigorous stirring. At the same time, prepolymer reacts with H_2_O, PEG, and TMP to produce CO_2_, urea, and urethane linkages in the presence of catalyst, causing the formation of polyurethane and small pores in the PRC. Due to the strong physical and chemical interaction between polyurethane and red mud, the red mud particles are tightly embedded into polyurethane during the curing process. Thus, a three-dimensional network structure is formed eventually.

**Figure 5:**
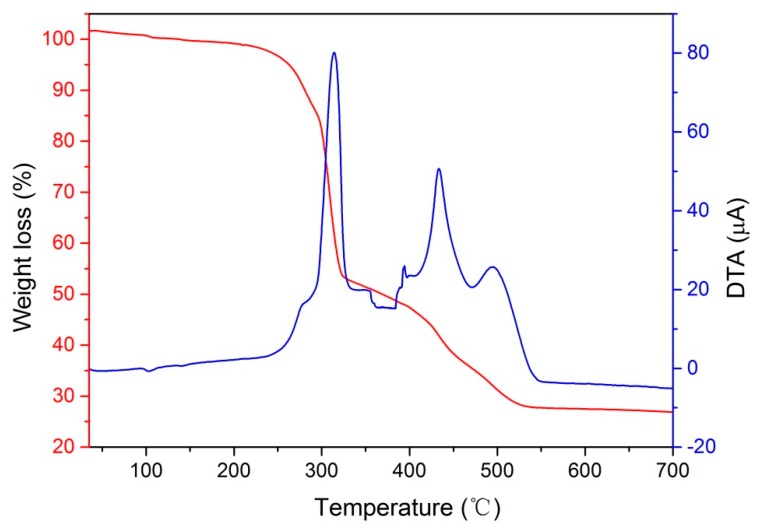
Thermal stability of PRC.

Thermal stability of PRC was investigated by TG-DTA. As presented in Figure 5, thermo gravimetric of PRC involves two stages. During the first stage, the mass of PRC showed a sharp decrease when the temperature was varied from 238 °C to 338 °C. The maximum weight loss rate was at 314 °C with the weight loss of 48.4%. The weight loss for PRC is due to the splitting and oxidation decomposition of organic compounds. In the second stage, the mass of PRC decreased gradually with increase of temperature during the temperature from 338 °C to 550 °C. Note that the weight loss reached 72.5% and the maximum weight loss rate was observed at 433 °C. The weight loss in this stage can be attributed to the carbonization of organic compounds. When the temperature was above 550 °C, no weight loss was observed. According to the characteristic temperatures in TG-DTA curves, it can be seen that the PRC appears to be thermally stable below 238 °C.

**Figure 6:**
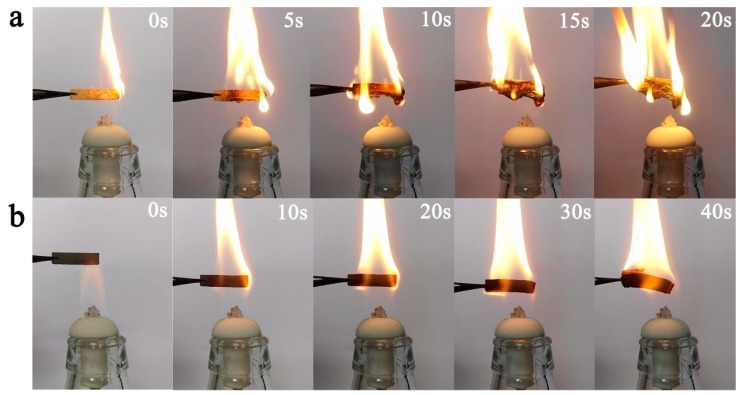
Combustion progress of polyurethane (**a**) and PRC (**b**) in air.

To better understand the fire-resistant performance of PRC, the combustion progress of polyurethane and PRC in air was investigated. As shown in Figure 6, an alcohol lamp was used to heat polyurethane and PRC directly. Polyurethane was almost completely burned in 20 s. In contrast, the PRC held its shape under combustion for 40 s. The combustion contrast of the polyurethane and PRC verified the good fire-resistant performance of the PRC, which can be attributed to the intrinsic non-flammability and uniform distribution of red mud particles in the PRC.

**Figure 7:**
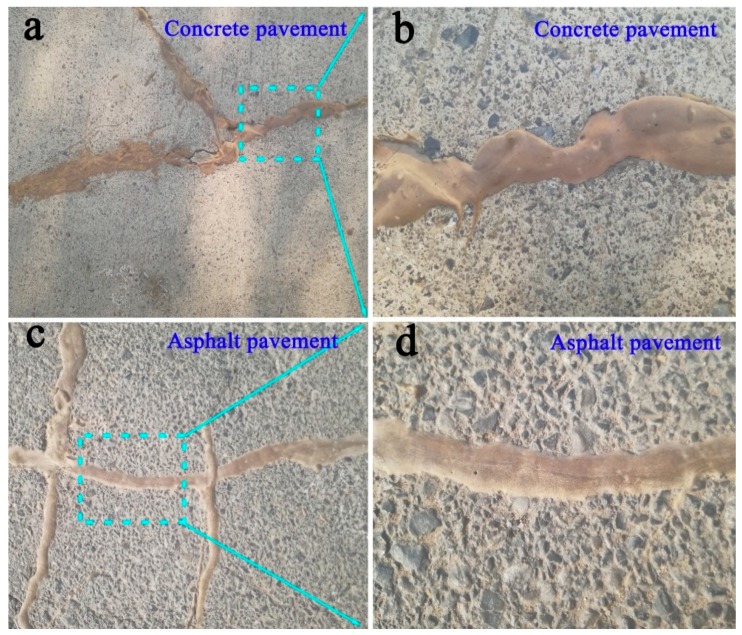
The application of PRC for crack repair of roadway. (**a**,**b**) Concrete pavement, (**c**,**d**) Asphalt pavement.

The synthetic PRC grouting material with excellent flexibility, stretchability. and flame retardancy was applied for the repairment of roadway cracks, and positive feedbacks have been obtained from observations on spot. As seen in Figure 7, the grouting material could permeate into tiny cracks of cement and asphalt roads through pump injection, and the fractured zone was integrated into a whole, resulting in the reinforcement of geologic defects or weakness in the roadway pavement. Up to now, no gap and other pavement damage appeared in these repairment zones, indicating a favorable repairment performance of PRC grouting material.

## 4. Conclusions

In this study, a PRC grouting material with flexibility, stretchability, and flame retardancy has been successfully prepared via a facile room-temperature-cured process. The resultant PRC grouting material possessed a three-dimensional network structure and satisfactory mechanical performance, the compressive strength at 50% compression state, and the fracture elongation could reach 29.2 MPa and 15.1%, respectively. FTIR and XRD analyses indicated that the main components of PRC grouting material were amorphous polyurethane and crystalline red mud. The combustion test verified that PRC had better fire-resistant performance than neat polyurethane. The obtained PRC grouting material was thermally stable below 238 °C, and had potential applications in wide fields (etc. roadway, bridge, underground engineering).

## Supplementary Materials

The following supplementary materials are available online at http://www.mdpi.com/2073-4360/10/8/906/s1.
