# Supplementary material for: Polyurethane/Red Mud Composites with Flexibility, Stretchability, and Flame Retardancy for Grouting
Source: Polymers (Basel). 2018 Aug 12;10(8):906. doi: 10.3390/polym10080906 (PMC6403916; doi:10.3390/polym10080906)
Supplement: Supplementary file 1 [file polymers-10-00906-s001.pdf]

Supplementary Material

Polyurethane/Red Mud Composites with Flexibility, Stretchability and Flame Retardancy for Grouting

Chunjing Zhang [1], Bo Shuai [1], Xuefeng Zhang [1], Xinxin Hu [1], Hui Zhang [2,*], Yuanheng Jia [1], Zhengpeng Yang [1,*] and Xuemao Guan [1]

[1] Institute of Materials Science and Engineering, Henan Polytechnic University, Jiaozuo 454000, China; zcj@hpu.edu.cn (C.Z.); boshuai@hpu.edu.cn (B.S.); xfzhang@163.com (X.Z.); xxhu@163.com (X.H.); yhjia@163.com (Y.J.); guanxuemao@163.com (X.G.)

[2] School of Energy and Engineering, Henan Polytechnic University, Jiaozuo 454000, China

***** Correspondence: huizhang@163.com or zhengpengyang2013@163.com; Tel.: +86-391-3989020

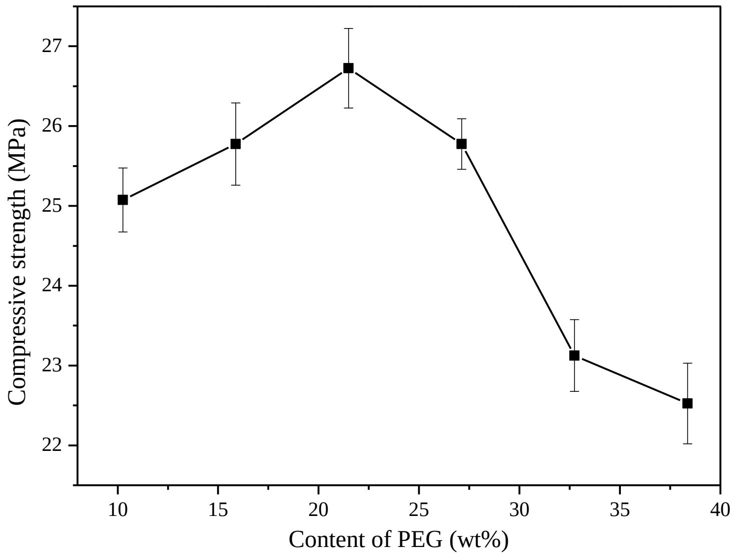

Figure S1. Effect of PEG content on the compressive strength of PRC. Average of three measurements (mean ± S.D.)

Our investigation indicated that the aqueous suspension of water and red mud with weight ratio of 38:113 possessed good stability and fluidity, and could be used as grouting material. Figure S1 shows the effect of PEG content on the compressive strength of PRC at 50% compression state. The compressive strength of PRC varied from to 25.08 to 26.73 MPa with the increase of PEG content from 10.3 to 21.5%, and then decreased sharply. Such phenomena can be explained as follows. The low PEG content will inhibit the polymer reaction of composite solution and the high PEG content will cause the presence of extra bobbles in cured PRC, resulting in a decreased toughness. Effect of TMP content on the compressive strength of PRC was also studied. As shown in Figure S2, the optimal TMP content was 2.5%.

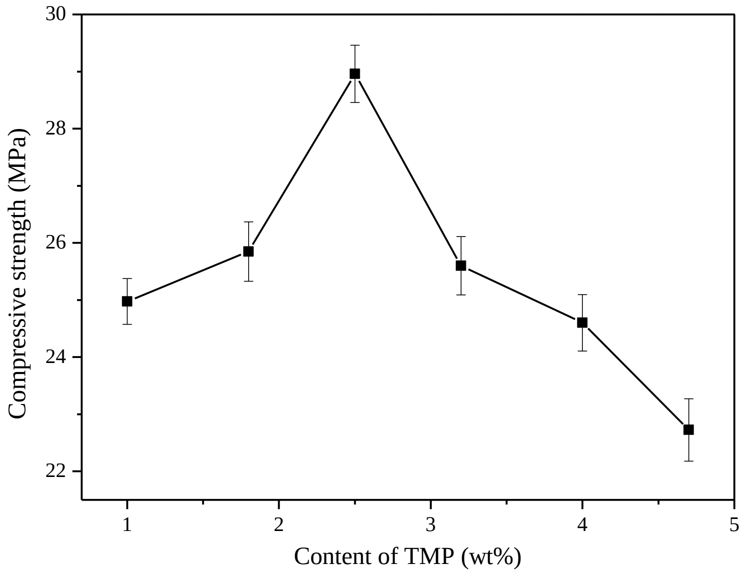

Figure S2. Effect of TMP content on the compressive strength of PRC. Average of three measurements (mean ± S.D.)
